# Novel computed tomographic chest metrics to detect pulmonary hypertension

**DOI:** 10.1186/1471-2342-11-7

**Published:** 2011-03-29

**Authors:** Andrew L Chan, Maya M Juarez, David K Shelton, Taylor MacDonald, Chin-Shang Li, Tzu-Chun Lin, Timothy E Albertson

**Affiliations:** 1 Division of Pulmonary/Critical Care and Sleep Medicine, University of California, Davis Medical Center, Sacramento, CA and VA Northern California Health Care System, USA; 2 Department of Radiology, University of California, Davis Medical Center, Sacramento, CA, USA; 3 Department of Public Health Sciences, Division of Biostatistics, University of California, Davis, CA, USA; 4 Department of Statistics, University of California, Davis, CA, USA

## Abstract

**Background:**

Early diagnosis of pulmonary hypertension (PH) can potentially improve survival and quality of life. Detecting PH using echocardiography is often insensitive in subjects with lung fibrosis or hyperinflation. Right heart catheterization (RHC) for the diagnosis of PH adds risk and expense due to its invasive nature. Pre-defined measurements utilizing computed tomography (CT) of the chest may be an alternative non-invasive method of detecting PH.

**Methods:**

This study retrospectively reviewed 101 acutely hospitalized inpatients with heterogeneous diagnoses, who consecutively underwent CT chest and RHC during the same admission. Two separate teams, each consisting of a radiologist and pulmonologist, blinded to clinical and RHC data, individually reviewed the chest CT's.

**Results:**

Multiple regression analyses controlling for age, sex, ascending aortic diameter, body surface area, thoracic diameter and pulmonary wedge pressure showed that a main pulmonary artery (PA) diameter ≥29 mm (odds ratio (OR) = 4.8), right descending PA diameter ≥19 mm (OR = 7.0), true right descending PA diameter ≥ 16 mm (OR = 4.1), true left descending PA diameter ≥ 21 mm (OR = 15.5), right ventricular (RV) free wall ≥ 6 mm (OR = 30.5), RV wall/left ventricular (LV) wall ratio ≥0.32 (OR = 8.8), RV/LV lumen ratio ≥1.28 (OR = 28.8), main PA/ascending aorta ratio ≥0.84 (OR = 6.0) and main PA/descending aorta ratio ≥ 1.29 (OR = 5.7) were significant predictors of PH in this population of hospitalized patients.

**Conclusion:**

This combination of easily measured CT-based metrics may, upon confirmatory studies, aid in the non-invasive detection of PH and hence in the determination of RHC candidacy in acutely hospitalized patients.

## Background

Pulmonary hypertension (PH) is characterized by the presence of increased pulmonary vascular resistance caused by a combination of vasoconstriction, vascular remodeling, and thrombosis. Unfortunately, it can be potentially life-threatening as progressive right ventricular dilatation and hypertrophy may lead to heart failure within a few years [1,2]. As the treatment of PH has advanced dramatically over the past decade[3], early diagnosis may be key to its optimal treatment. While right heart catheterization (RHC) remains the "gold standard" for the measurement of pulmonary arterial pressure (PAP)[4], its invasive nature confers both risk and expense, and hence can delay diagnosis. Echocardiography as a noninvasive means of estimating PAP is limited in patients with obesity, lung hyperinflation and pulmonary fibrosis [5-7]. Magnetic resonance imaging methods have unfortunately also not been shown to accurately estimate PAP[8].

Other noninvasive PH screening tools include a prediction formula for estimating mean PAP using standard pulmonary function measurements in patients with idiopathic pulmonary fibrosis [9]. In addition, computed tomography (CT)-determined main pulmonary artery diameter has been shown to have excellent diagnostic value in the detection of PH in patients with parenchymal lung disease[10]. Such noninvasive approaches towards the detection of PH can reduce patient risk and expense, and may allow earlier patient screening towards a confirmatory RHC [11] or perhaps even obviate its necessity. This study retrospectively reviewed the records of inpatients who had undergone a RHC together with a CT chest. A pre-defined set of CT chest-based metrics was then measured, and the relationship of these metrics to RHC-demonstrated PH was assessed.

## Methods

### Patients

The medical records of 101 hospitalized adult patients who consecutively underwent chest CT with or without contrast and a resting RHC during the same hospitalization were retrospectively reviewed. These patients had been admitted to this tertiary care teaching institution between January 2006 and July 2006. Approval for this review was obtained from the University of California, Davis Institutional Review Board with waiver of consent.

### Measurements

Non-ECG-gated CT scans of the chest were performed using GE Lightspeed 16 scanners (GE Medical Systems; Milwaukee, Wisconsin). Our standard reconstruction protocol utilized helical technique, 5 mm slice every 5 mm with 1.25 mm every 1.25 mm reconstruction as well. The lung windows were also reconstructed with a "bone algorithm" and the soft tissue windows were reconstructed with a standard soft tissue smoothing algorithm. Standard lung windows (Width 1850, Level -740) on bone reconstruction algorithms and standard soft tissue windows (Width 400, Level 80) were used.

Two separate teams, each consisting of a radiologist and a pulmonologist, blinded to the clinical and hemodynamic data, independently reviewed the chest CT's. Pre-defined radiographic metrics corresponding to possible predictors of PH and to potential indicators of body habitus (to standardize predictors of PH) were measured by each team member and then averaged (Table 1).

**Table 1:** Radiographic metrics

Hypothesized predictors of PH	How measured
Main pulmonary artery diameter (PA)	Widest lumen at or near level of PA bifurcation *

Right pulmonary artery diameter (RPA)	Widest lumen caudal to ascending aorta

Left pulmonary artery diameter (LPA)	Widest lumen

Right descending pulmonary artery (RDPA)	Distance from lateral wall of right bronchus intermedius to lateral wall of RDPA (equivalent to the RDPA measurement on a chest x-ray)

True right descending pulmonary artery (true RDPA)	RDPA lumen diameter only *

True left descending pulmonary artery (true LDPA)	Lumen diameter distal to left upper lobe bronchus takeoff

Right ventricular free wall (RV wall)	Mid-ventricle *

Right ventricular lumen diameter (RV lumen)	Mid-ventricle *

Left ventricular free wall (LV wall)	Mid-ventricle *

Left ventricular lumen diameter (LV lumen)	Mid-ventricle *

IV septum bowing into LV	Yes or no

Left apical artery to corresponding bronchus ratio	Most apical bronchovascular pair

Hilar diameter (HD)	At level of right middle lobe bronchus takeoff

Hilar/Thoracic ratio	HD/Inner thoracic diameter (TD) measured at same level as HD

**Landmarks used to standardize predictors of PH**	

Ascending aorta diameter (AA)	Widest diameter at the level of the PA measurement *

Descending aorta diameter (DA)	At same level as AA *

* Also see Figure 1

Cardiac catheterization was performed at rest for clinical indications. Hemodynamic measurements, including mean pulmonary arterial pressure (mPAP), pulmonary wedge pressure (PWP), patient diagnoses and demographics (sex, age, height, and weight) were recorded. Body surface area (BSA) was calculated using the formula BSA = (W^0.425 ^× H^0.725^) × 0.007184, and body mass index (BMI) was calculated using the formula BMI = weight (kg)/(height (cm))^2^.

PH was defined as a resting mPAP of 25 mmHg or greater; "no-PH" was defined as mPAP <25 mmHg.

### Statistical Analyses

Summary statistics are reported as mean ± standard deviation (median; range). A two-sided Wilcoxon rank-sum test was used to compare each of the quantitative hypothesized predictors of PH and various demographics between the PH and no-PH groups. A two-sided Fisher's exact test was used to compare gender, obesity (BMI ≥30), and proportion of mechanical ventilation between the PH and no-PH groups. Statistical analyses involving the RV wall, RV lumen, LV wall, LV lumen, or interventricular septum were performed using only data derived from CT's that were contrast-enhanced.

Simple logistic regression was used to study the relationship between each hypothesized predictor of PH and the outcome PH vs. no-PH. The optimal cutoff point or upper limit of normal (ULN) for the quantitative hypothesized predictor of PH was determined using receiver operating characteristic (ROC) analysis, where the ULN was deemed to be the value that yielded the best trade off between sensitivity and specificity for each PH predictor. Multiple logistic regression was used to assess the relationship between each of the dichotomous hypothesized predictors of PH (i.e. variables dichotomized at the ULN cutoff) and the outcome PH vs. no-PH in order to control for sex, ascending aorta diameter (AA), BSA, thoracic diameter (TD), and pulmonary wedge pressure (PWP) >15. A p-value ≤ 0.05 was considered statistically significant. Statistical analyses were performed with SAS v 9.2 (SAS Institute Inc., Cary, NC, USA).

## Results

101 consecutive hospitalized patients (46 women, 55 men) who underwent both CT chest and RHC were included. Their mean age was 61.4 ± 15.6 years (median = 60; range 23 to 91 years). Fifty-three patients had PH. Fifty-seven percent of patients with PH, and 52% of the no-PH patients were male. The underlying patient primary diagnoses reflect the reason for hospitalization, and were heterogeneous (Table 2).

**Table 2:** Patient primary diagnoses

	**N**
Coronary arterial disease	27
Congestive heart failure	22
Valvular disease	12
Pulmonary embolism	5
Cardiac arrhythmia	4
Pulmonary infection	4
Aortic aneurysm or dissection	3
Idiopathic PH	3
Interstitial lung disease	3
ARDS	2
Cardiac tamponade	2
COPD	2
Obstructive sleep apnea	2
Other *	10

*Other includes: cancer, anorexigen drug use, cerebrovascular accident, end-stage renal disease, hepatopulmonary syndrome, kyphoscoliosis, prior history of lung transplant, malignant hypertension, scleroderma, and trauma.

The RHC's and chest CT's were performed a mean of 3 days apart (median = 1 day, range = 0-16 days); 46% of RHC's were performed on the same day as the chest CT's, and most within 2 days (60%). A majority of CT's were contrast-enhanced (36/48 in the no-PH group and 41/53 in the PH group). Overall, 43% (43/101) of patients had an elevated PWP (>15 mmHg), and most were in the PH group (40/53 = 75%).

There was no significant difference in age, height, or sex between the PH and no-PH groups. However, PH patients had a significantly higher mean weight, BSA, and BMI than no-PH patients (Table 3). 41% of PH patients were obese (BMI≥30) compared to 18% of no-PH patients (p = 0.0175).

**Table 3:** Patient demographics

	PH mean ± SD	No-PH mean ± SD	P
Age (yrs)	59.5 ± 15.4	63.5 ± 15.8	0.1948
Sex (% men)	56.6	52.1	0.6923
Height (cm)	169.8 ± 10.9	167.4 ± 10.8	0.3395
**Weight (kg)**	**85.4 ± 22.1**	**71.7 ± 18.5**	**0.0029**
**BSA**	**1.9 ± 0.3**	**1.8 ± 0.3**	**0.0085**
**BMI**	**29.6 ± 7.1**	**25.3 ± 4.9**	**0.0024**
Mechanical ventilation (%)	15.1	8.3	0.3650

A comparison of the predictors of PH revealed significantly higher measurements of PA, RDPA, true RDPA, and hilar diameters in the PH group. The RV free wall thickness, RV wall/LV wall ratio, hilar diameter, and main PA/AA ratio were also significantly increased in this group (Table 4). Inter-observer variability in measurements within each team was less than 5%.

**Table 4:** Comparison of hypothesized predictors of PH in the PH and no-PH groups

	PH mean ± SD	No-PH mean ± SD	P
**Main PA diameter (mm)**	**32.2 ± 5.3**	**29.0 ± 3.9**	**0.0021**
Left PA diameter (mm)	24.2 ± 4.6	22.9 ± 3.2	0.1225
Right PA diameter (mm)	24.0 ± 4.3	23.4 ± 3.7	0.4618
**RDPA diameter (mm)**	**19.4 ± 4.4**	**17.0 ± 2.6**	**0.0072**
**True RDPA diameter (mm)**	**15.3 ± 3.7**	**13.4 ± 2.4**	**0.0027**
True LDPA diameter (mm)	19.4 ± 3.8	18.1 ± 3.4	0.0509
**RV free wall thickness (mm)**	**5.4 ± 2.4**	**4.0 ± 1.2**	**0.0023**
RV lumen diameter (mm)	36.9 ± 10.1	35.3 ± 7.9	0.4293
LV free wall (mm)	11.6 ± 3.6	11.1 ± 3.3	0.4820
LV lumen (mm)	44.0 ± 15.5	42.9 ± 9.5	0.9942
**Hilar diameter (mm)**	**127.1 ± 13.7**	**118.2 ± 12.0**	**0.0018**
**RV wall/LV wall ratio**	**0.51 ± 0.25**	**0.38 ± 0.14**	**0.0214**
RV lumen/LV lumen ratio	1.02 ± 0.85	0.85 ± 0.26	0.9796
L apical artery/bronchus ratio	1.33 ± 0.43	1.27 ± 0.35	0.6023
Hilar/thoracic ratio	0.51 ± 0.04	0.49 ± 0.04	0.0687
**Main PA/AA ratio**	**0.97 ± 0.2**	**0.86 ± 0.13**	**0.0014**
Main PA/DA ratio	1.24 ± 0.27	1.15 ± 0.17	0.2023
			
	PH %	no-PH %	
IV septum bowed into LV	16.7	2.7	0.0608

See table 1 for definitions of radiographic metrics.

### Relationship of hypothesized predictors of PH to the dichotomous outcome PH vs. no-PH

The seven significant predictors of PH in Table 4 were also found to be significantly correlated to the outcome (PH vs. no-PH) when using logistic regression. An OR and ULN for each predictor of PH was determined from these regression analyses. The optimal cutoff point or ULN was determined by the ROC analysis to be the value that yielded the best tradeoff between sensitivity and specificity for each PH quantitative predictor (Table 5).

**Table 5:** Simple logistic regression of hypothesized predictors of PH to outcome (PH vs. no-PH)

	P	OR	Lower 95% CI	Upper 95% CI	ROC AUC	Sensitivity (%)	Specificity (%)	Upper limit of normal (ULN) cutoff
**Main PA diameter (mm)**	**0.0020**	**1.2**	**1.1**	**1.3**	**0.68**	**67.9**	**56.3**	**29 mm**
Left PA diameter (mm)	0.1158	1.1	1.0	1.2	0.59	47.2	62.5	24 mm
Right PA diameter (mm)	0.4187	1.0	0.9	1.1	0.54	37.7	64.6	25 mm
**RDPA diameter **(mm)	**0.0031**	**1.2**	**1.1**	**1.4**	**0.66**	**43.4**	**79.2**	**19 mm**
**True RDPA diameter **(mm)	**0.0053**	**1.2**	**1.1**	**1.4**	**0.67**	**32.1**	**83.3**	**16 mm**
True LDPA diameter (mm)	0.0814	1.1	1.0	1.2	0.61	32.1	77.1	21 mm
**RV Free Wall thickness **(mm)	**0.0070**	**1.5**	**1.1**	**2.1**	**0.69**	**21.4**	**91.9**	**6 mm**
RV Lumen diameter (mm)	0.4248	1.0	1.0	1.1	0.55	66.7	18.9	30 mm
LV Free Wall (mm)	0.5105	1.0	0.9	1.2	0.55	9.5	78.4	15 mm
LV Lumen (mm)	0.7136	1.0	1.0	1.0	0.50	0.0	83.3	57 mm
**Hilar diameter **(mm)	**0.0017**	**1.1**	**1.0**	**1.1**	**0.68**	**54.7**	**68.8**	**124 mm**
**RV wall/LV wall ratio**	**0.0152**	**22.8**	**1.8**	**283.4**	**0.64**	**61.9**	**64.9**	**0.32**
RV lumen/LV lumen ratio	0.2942	1.8	0.6	5.3	0.50	19.0	94.4	1.28
L apical artery/broncus ratio	0.4379	1.5	0.5	4.2	0.53	11.3	87.5	1.75
Hilar/thoracic ratio	0.0785	>5000	0.4	>5000	0.61	35.8	79.2	0.52
**Main PA/AA ratio**	**0.0035**	**58.7**	**3.8**	**900.7**	**0.68**	**79.2**	**50.0**	**0.84**
Main PA/DA ratio	0.0626	6.7	0.9	50.3	0.57	30.2	83.3	1.29

See table 1 for definitions of radiographic metrics.

### Controlling for body size, age, sex and PWP

**Figure 1:**
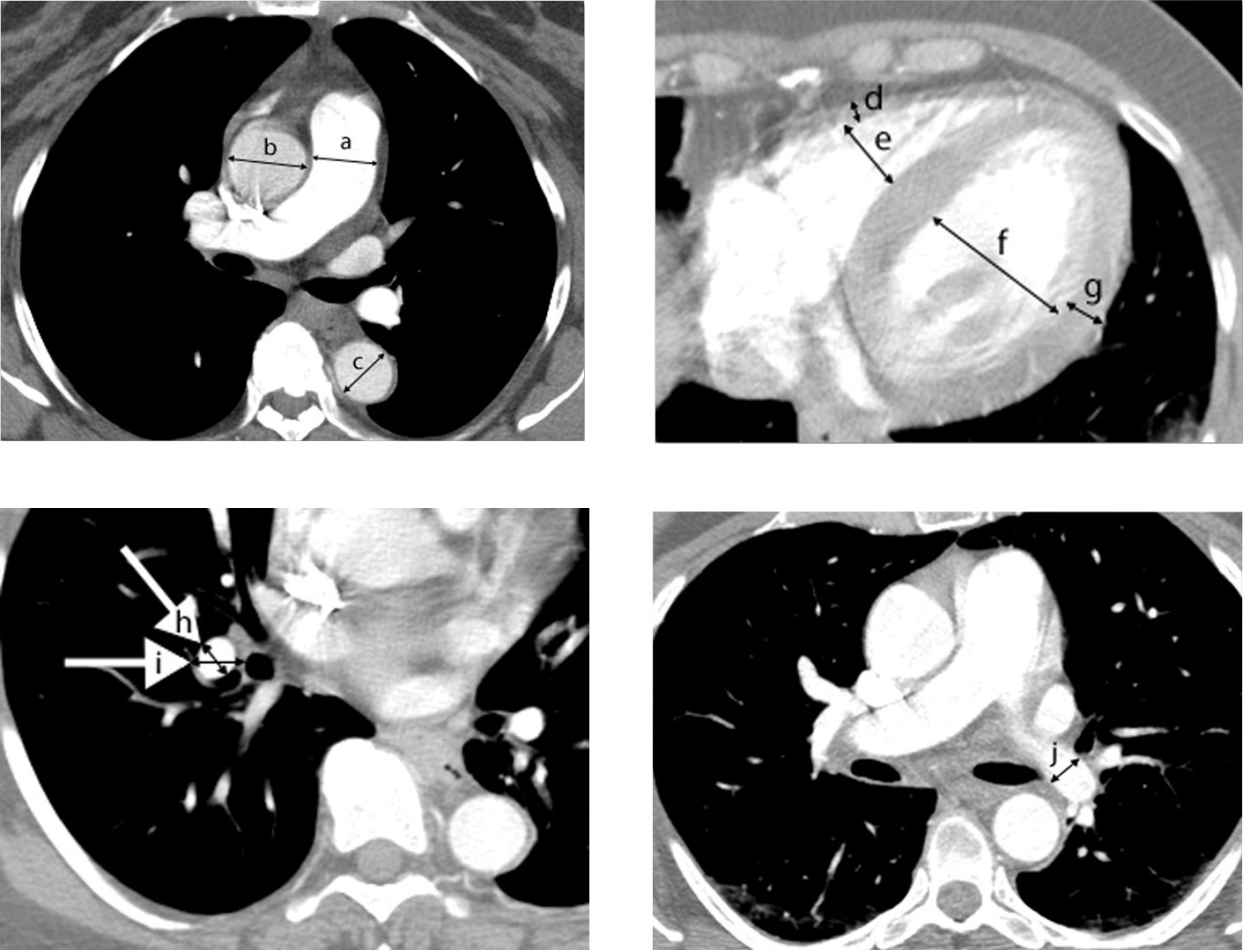
**Radiographic measurements**. The radiometric measurements used to derive the predictors of PH that were found to be significant in Table 6 included: main PA (a), AA (b), DA (c), RV free wall (d), RV lumen (e), LV lumen (f), LV free wall (g), true RDPA (h), RDPA as would be seen on chest x-ray (i), and true LDPA (j).

Each dichotomous CT-derived predictor of PH (e.g. PA diameter ≥ 29 mm) and potential confounders (age, sex, AA, BSA, thoracic diameter, and PWP category (>15 or ≤ 15 mmHg)) were regressed to the outcome, PH vs. no-PH, using multiple logistic regression models. In addition to the parameters in Table 5, two additional predictors of PH (interventricular (IV) septum bowing into LV and main PA/AA ratio > 1) were included in these analyses. Several predictors of PH were found to be statistically significant when controlling for age, sex, PWP, and indicators of body size (Table 6). Diagrammatic representations of the significant predictors of PH from Table 6 are represented in Figure 1.

**Table 6:** Multiple logistic regression to control for potential confounders

	P	OR	AUC	Sensitivity (%)	Specificity (%)
**Main PA diameter ≥29 mm**	**0.0196**	**4.8**	**0.93**	**77.4**	**89.6**
Left PA diameter ≥24 mm	0.2160	2.6	0.92	77.4	87.5
Right PA diameter ≥25 mm	0.4461	1.9	0.91	73.6	93.8
**RDPA diameter **≥**19 mm**	**0.0059**	**7.0**	**0.93**	**83.0**	**85.4**
**True RDPA diameter **≥**16 mm**	**0.0487**	**4.1**	**0.92**	**83.0**	**87.5**
**True LDPA diameter **≥**21 mm**	**0.0075**	**15.5**	**0.93**	**79.2**	**91.7**
**RV free wall **≥**6 mm**	**0.0303**	**30.5**	**0.95**	**81.0**	**91.9**
RV lumen ≥30 mm	0.0915	5.8	0.95	92.9	73.0
LV free wall ≥15 mm	0.1607	5.2	0.95	85.7	83.8
LV lumen ≥57 mm	0.3945	3.0	0.94	76.2	88.9
Hilar diameter ≥124 mm	0.2968	2.2	0.92	81.1	75.0
**RV wall/LV wall ratio **≥**0.32**	**0.0141**	**8.8**	**0.96**	**78.6**	**83.8**
**RV lumen/LV lumen ratio **≥**1.28**	**0.0196**	**28.8**	**0.95**	**85.7**	**86.1**
L apical artery/bronchus ratio ≥1.75	0.2851	3.5	0.92	75.5	87.5
Hilar/thoracic ratio ≥0.52	0.0757	3.7	0.92	75.5	87.5
**Main PA/AA ratio **≥**0.84**	**0.0208**	**6.0**	**0.93**	**73.6**	**91.7**
**Main PA/DA ratio **≥**1.29**	**0.0269**	**5.7**	**0.93**	**77.4**	**89.6**
IV septum bowing into LV (yes or no)	0.1053	11.6	0.95	81.0	89.2
**Main PA/AA ratio >1**	**0.0085**	**9.1**	**0.93**	**86.8**	**79.2**

Regression of the outcome variable (PH vs. no-PH) to the predictors:

ULN for each hypothesized predictor of PH, age, sex, ascending aorta diameter (AA), BSA, thoracic diameter (TD), and pulmonary wedge pressure (PWP) >15 mmHg.

Note: one logistic regression model was created for each hypothesized predictor of PAH. For example, the analysis in the first row above included the predictors: Main PA diameter ≥29, age, sex, AA, BSA, TD, and PWP >15. See table 1 for definitions of radiographic metrics.

## Discussion

A National Institutes of Health Registry found that the mean time from onset of symptoms (dyspnea 60%, fatigue 19%, syncope 8%, and chest pain 7%) to correct diagnosis of PH was 2 years[12]. Early diagnosis is key to effective treatment and potential prevention of further vascular remodeling. When ineffectively treated, the median survival of patients with idiopathic PH is 2.8 years [13]. However over the past 2 decades, PH treatment options have evolved to improve both survival and quality of life [14]. This retrospective analysis suggests that CT-based metrics can help detect PH, potentially enabling earlier treatment.

Although no significant differences in age, height or gender were found between PH and no-PH patients in this study, patients in the PH group demonstrated significantly greater body weight, BMI and BSA. There were significantly more obese patients in the PH group (41%) compared to the no-PH group (18%). Nevertheless, most patients in both groups were not obese. The presence of obesity, though, may contribute to PH, as Hague et al [15] found pulmonary hypertensive changes in 72% of obese subjects, a statistically higher proportion than when compared to the control group (p < 0.001).

Despite the heterogeneity of the primary diagnoses of patients in this study, the commonest diagnoses of coronary arterial disease (CAD) and congestive heart failure (CHF) were approximately equal in preponderance in both the PH group and the no-PH group (47% vs. 50%, respectively). A majority of patients (76%) demonstrated an elevated PWP in the PH group, compared to 6% in the no-PH group. This may be due in part to the higher rate of obesity in the PH group, and possible development of obesity cardiomyopathy, in the absence of other risk factors such as CAD[16].

The current study not only confirmed a significant difference in the PA diameter between PH and no-PH patients, but also showed significant differences in measurement of the RDPA, True RDPA, RV free wall thickness, RV Wall/LV Wall ratio, Hilar Diameter, and Main PA/AA ratio (Table 4). These pre-defined CT-based parameters had been previously selected at least in part on the basis of the existing published literature[17-20], modified and augmented using straightforward to measure CT-based metrics. For example, the width of the RDPA has been shown to be a significant predictor for PH [18,20], as has the diastolic RV outflow tract wall thickness [21]. In addition to this, this study novelly found a significant difference between the PH group and no-PH group in terms of the true RDPA diameter. Certainly, the presence of significant abnormalities in the measurements above ought to promptly engender further investigation as to the presence of pulmonary hypertension.

Modeling to control for the potential confounders[22-24] of age, sex, AA, BSA, thoracic diameter, and especially PWP (>15 or ≤15 mmHg) using multiple logistic regression, showed that 10 parameters were significant predictors of PH, despite the fact that 76% of patients in the PH group had an elevated PWP, and 30.7% of all patients were obese (Table 6).

Beiderlinden et al, in a study of ARDS patients with at least moderate PH (mean PA pressure of >30 mmHg), reported a sensitivity of 54% and a specificity of 63% utilizing a pulmonary artery trunk diameter ≥29 mm[25]. They also suggested that CT chest parameters were an unreliable tool in the detection of PH in ARDS patients; speculating that pulmonary vascular changes in chronic rather than acute PH may lead to remodeling of the PA and hence enlargement of its diameter. While it is difficult to ascertain the proportion of patients in the current study with chronic PH, controlling for age, sex, AA, BSA, thoracic diameter, and PWP category yielded a superior sensitivity and specificity for prediction of PH of 77.4% and 89.6%, respectively using an ULN value for PA diameter of ≥29 mmHg.

The current study also found that an ULN cutoff of 1.29 for the main PA/DA ratio and an ULN cutoff of >0.84 for the main PA/AA ratio could both be used to predict PH. While this main PA/AA ratio had been demonstrated in a previous study to strongly correlate with mPAP in a patient population under 50 years of age also with heterogenous diagnoses[23], a more recent study however has suggested that the traditional PA/AA ratio >1 is a poor diagnostic tool as it includes normal patients and is negatively affected by age [26]. In contrast, our study found significance using a main PA/AA ratio of ≥0.84 or >1 in acutely ill patients even after controlling for age in detecting the presence of PH.

Other novel predictors of PH that were found to be significant in this study included specific cardiac measurements, particularly the RV free wall of ≥6 mm, RV lumen/LV lumen ratio ≥1.28, and RV wall/LV wall ratio ≥0.32. Of these, the RV lumen/LV lumen ratio ≥1.28 showed high sensitivity (85.7%) and specificity (86.1%, OR = 28.8). Both the true LDPA diameter ≥21 mm and the true RDPA diameter ≥16 mm also afforded good sensitivities (79.2% and 83%, respectively) and high specificities (91.7% and 87.5%, respectively), with OR's of 15.5 and 4.1.

An ULN cutoff of ≥6 mm for the RV Free Wall showed significant promise as a predictor of PH (p = 0.0303) with a high OR of 30.5, and sensitivity of 81% and specificity of 91.9%. It has been suggested that the right ventricle adapts to the increased afterload in PH by increasing muscle mass and hence wall thickness, and by assuming a more rounded shape[27]. Other investigators[28] studied 16 patients with primary pulmonary hypertension and found an increase in resting right ventricular mass. Cardiovascular Magnetic Resonance Imaging may be helpful to further assess right ventricular structure and function in PH patients [29]. PA volume estimation utilizing CT-volumetry may also be useful in PH detection[30].

The current study has a number of limitations in part due to its retrospective nature. Selection bias may have been introduced by only including patients who underwent both RHC and chest CT. Nevertheless, a consecutive cohort of acutely hospitalized patients with heterogeneous diagnoses were studied, of whom about half had an acute primary diagnosis of CAD or CHF. While the majority of patients in the PH group were associated with an elevated PWP, controlling for this using multiple logistic regression models still resulted in statistical significance for eight pre-defined CT chest metrics for detecting PH.

Additional limitations include the fact that some patients underwent CT scanning breathing spontaneously, whilst others were on positive pressure ventilation. However, this was limited to a minority of patients (only 8.3% in the no-PH group and 15.1% in the PH group). Positive pressure ventilation may have affected end-expiratory PA diameter due to varying intrathoracic pressures and lung volumes, affecting transmural PA pressure. In a secondary analysis of the non-mechanically ventilated patient cohort (n = 89), all significant findings reported in Table 6 retained their significance, except for three parameters (true RDPA diameter ≥16 mm, RV wall/LV wall ratio ≥0.32, and RV lumen/LV lumen ratio ≥1.28). The loss of significance in these three parameters is unclear, and may have been related to a smaller sample size, a loss of power or physiological reasons.

The RHC's and chest CT's were performed a mean of 3 days apart (median = 1 day), another limitation. It is acknowledged that significant changes in PWP and hence PAP can occur even on an hourly or daily basis, depending on treatment. Nevertheless, relatively short delays between measurements are likely to only result in small and randomly distributed errors[25].

Standard CT window widths were used in this study, thus minimizing variability in anatomic measurements when compared to using non-standard window widths via a computer program using density profiles[22]. Clinician bias in measuring parameters was limited by using separate teams, blinded to the clinical and hemodynamic data, to independently review the chest CT's.

The high incidence of CT contrast enhancement in 75% of the no-PH group and in 77% of the PH group may have aided in metric measurement. Nevertheless, others have reported good inter-observer measurement accuracy in a study of a heterogeneous group of patients with PH, utilizing a mixture of enhanced and unenhanced CT scans of the chest[23]. Edwards et al also demonstrated that the measurement of the pulmonary artery diameter was extremely reproducible using unenhanced CT scans, with a standard deviation for the difference between 2 measurements of less than 0.08 cm and a mean difference of only 0.02 cm[22].

## Conclusions

This study has shown that there is a group of CT chest-derived predictors of PH that shows significance, even after controlling for age, sex, AA, BSA, thoracic diameter, and especially PWP. Novel predictors including RV free wall ≥ 6 mm, RV lumen/LV lumen ratio ≥ 1.28, True LDPA diameter ≥ 21 mm and True RDPA diameter ≥ 16 mm amongst others, may serve as a template to detect PH in such patients with acute illnesses requiring hospitalization and aid in determining which patients require RHC. A confirmatory prospective multi-centre study utilizing the significant CT chest metrics above, and large enough to enable pre-defined subset analysis on the various WHO Groups of PH, is needed.

## Abbreviations

AA: ascending aorta; AUC: area under the curve; BMI: body mass index; BSA: body surface area; CAD: coronary arterial disease; CHF: congestive heart failure; CT: computed tomography; DA: descending aorta; IV: interventricular; LDPA: left descending pulmonary artery; LV: left ventricle; mPAP: mean pulmonary arterial pressure; OR: odds ratio; PA: pulmonary artery; PAP: pulmonary arterial pressure; PH: pulmonary hypertension; PWP: pulmonary wedge pressure; RDPA: right descending pulmonary artery; RHC: right-heart catheterization; ROC: receiver operating characteristic; RV: right ventricle; TD: thoracic diameter; TL: tracheal lumen; ULN: upper limit of normal.

## Competing interests

The authors declare that they have no competing interests.

## Authors' contributions

ALC: study concept and design; acquisition, analysis and interpretation of data; and drafting of manuscript. MMJ: study concept and design, coordination for the acquisition of data, analysis and interpretation of data, and drafting of manuscript. DKS: study concept and design; acquisition, analysis and interpretation of data; and critical revision of manuscript. TM: acquisition, analysis and interpretation of data; and critical revision of manuscript. CSL: analysis and interpretation of data; statistics expertise; and drafting of manuscript TCL: analysis and interpretation of data; statistics expertise; and drafting of manuscript. TEA: study concept and design; analysis and interpretation of data; and critical revision of manuscript.

## Pre-publication history

The pre-publication history for this paper can be accessed here:

http://www.biomedcentral.com/1471-2342/11/7/prepub
